# Personalized medicine—a modern approach for the diagnosis and management of hypertension

**DOI:** 10.1042/CS20160407

**Published:** 2017-11-06

**Authors:** Carmine Savoia, Massimo Volpe, Guido Grassi, Claudio Borghi, Enrico Agabiti Rosei, Rhian M. Touyz

**Affiliations:** 1 Clinical and Molecular Medicine Department, Cardiology Unit Sant’Andrea Hospital, Sapienza University of Rome, Rome, Italy; 2 IRCCS Neuromed, Pozzilli (Is), Italy; 3 Clinica Medica, Department of Medicine and Surgery, University Milano-Bicocca, Milan, IRCCS Multimedica, Sesto San Giovanni, Milan, Italy; 4 Chair of Internal Medicine, Department of Medical and Surgical Sciences, Faculty of Medicine, University of Bologna, Bologna, Italy; 5 Department of Clinical and Experimental Sciences, Clinica Medica, University of Brescia, Brescia, Italy; 6 Institute of Cardiovascular and Medical Sciences, British Heart Foundation Glasgow Cardiovascular Research Centre, University of Glasgow, Glasgow, U.K.

**Keywords:** compliance, hypertesion, personalized medicine, systems biology

## Abstract

The main goal of treating hypertension is to reduce blood pressure to physiological levels and thereby prevent risk of cardiovascular disease and hypertension-associated target organ damage. Despite reductions in major risk factors and the availability of a plethora of effective antihypertensive drugs, the control of blood pressure to target values is still poor due to multiple factors including apparent drug resistance and lack of adherence. An explanation for this problem is related to the current reductionist and ‘trial-and-error’ approach in the management of hypertension, as we may oversimplify the complex nature of the disease and not pay enough attention to the heterogeneity of the pathophysiology and clinical presentation of the disorder. Taking into account specific risk factors, genetic phenotype, pharmacokinetic characteristics, and other particular features unique to each patient, would allow a personalized approach to managing the disease. Personalized medicine therefore represents the tailoring of medical approach and treatment to the individual characteristics of each patient and is expected to become the paradigm of future healthcare. The advancement of systems biology research and the rapid development of high-throughput technologies, as well as the characterization of different –omics, have contributed to a shift in modern biological and medical research from traditional hypothesis-driven designs toward data-driven studies and have facilitated the evolution of personalized or precision medicine for chronic diseases such as hypertension.

## Introduction

Hypertension occurs in more than one billion individuals, and its prevalence appears to affect approximately 40% of the general population, with an increase upon aging from 7% in younger individuals (18–39 years old), to 65% in individuals over 59 years old [1]. Hypertension represents the most relevant risk factor for death and disability worldwide, causing an estimated 9.4 million deaths every year [2]. Hypertension contributes 3.5 times more to the total global disease burden of cardiovascular disease than smoking and 1.6 times that of hypercholesterolemia [3]. Prospective cohort studies have reported a continuous log-linear association between blood pressure and vascular events beginning at values of 115/75 mmHg with no apparent threshold [4–9]. The burden of hypertension and its associated arteriosclerotic target organ complications are increasing as the population increases in size, age, and obesity [10]; this association seems to exist across large and diverse population groups from several ethnicities, with and without established vascular disease [4,11,12]. Nevertheless, hypertension contributes to an unequaled burden of disease globally [13], for example, Western European countries exhibit a downward trend, in contrast with Eastern European countries, which show an increase in death rates from stroke [14,15].

Tissue and organ remodeling processes induced by hypertension may impair the physiology and structure of the heart, arteries, kidneys, and brain. Thus, the presentation of target organ complications in hypertensive patients may reflect different pathophysiological abnormalities including: diastolic and systolic dysfunction, left ventricular hypertrophy, congestive heart failure (39% of cases in men and 59% in women), coronary disease, accelerated atherosclerosis, aneurysm formation, stroke, nephrosclerosis, and renal failure [16]. Approximately 50% of hypertensive patients develop related end-organ damage if blood pressure is untreated or not treated to target. Yet hypertension remains the most common modifiable risk factor for cardiovascular disease [10]. The net benefit derived from antihypertensive therapy has been demonstrated in clinical trials and is associated with reduction in stroke (35–40%), myocardial infarction (20–25%), and in heart failure incidence (50%) [17–28]. In particular, a 10 mmHg reduction in systolic blood pressure is associated with a 22% reduction in coronary heart disease and 41% reduction in stroke, with a slight difference in reduction in cardiometabolic mortality for men (46%) and women (41%) [29]. Interestingly, mortalities attributable to heart attack and stroke are declining, whereas the incidence and prevalence of heart and kidney failure are rising [14]. This is mainly due to the therapeutic targeting of structural vascular alterations that lead to increased stiffness of large and small arteries in hypertensive patients, particularly if diabetes is associated [30–32].

For over a decade, international guidelines for the management of hypertension have stratified cardiovascular risk into different categories, based on blood pressure values, the presence of other cardiovascular risk factors, and organ damage [33,34–39]. Accordingly, patients at high or very high cardiovascular risk require intensive cardiovascular risk-reducing measures and eventually intensive drug therapy. Nonetheless, whether blood pressure lowering treatment reduces the risk of cardiovascular disease in all types of patient populations remains unclear, particularly in the elderly, individuals with lower blood pressure values, or with comorbidities [40–43]. Hence, although a modern drug-based therapeutic approach has the capacity to reduce blood pressure in a high percentage of patients with hypertension, the best approach to reduce blood pressure remains controversial [36,44–47].

Moreover, despite increased awareness of hypertension-related complications, reductions in major risk factors, widespread availability of very good antihypertensive drugs, as well as the better use of antihypertensive therapies [1,4,33,48], the control of blood pressure to target values is still poor (only 30% of treated patients achieve blood pressure control ≤140/90 mmHg), thus the overall prevalence of hypertension has increased by approximately 10% [1,49,50]. For instance, in a large Italian population of treated hypertensive patients followed for 10 years, it has been shown that approximately 60% of hypertensive patients were treated and among these only 33% achieved effective blood pressure control [51]. These data are similar to other reports from different countries. This is mainly attributable to reduced compliance and adherence to antihypertensive treatment [52–54], which is associated with a lack of improvement in cardiovascular risk. Among the explanations for the poor adherence we should consider the variability in the individual response to a given treatment, as well as the individual, distinctive, and not always predictable occurrence of adverse events related to antihypertensive medications, which are usually not life-threatening although frustrating for the patient who eventually may decide to discontinue the therapy. This can generate a ‘trial-and-error’ approach in clinical practice that is characterized by the switching to another medication in case the first therapeutic choice was not satisfactory after few weeks. This approach is based on general information derived mainly from data of randomized clinical trials regarding the drug that might work for that particular patient. In clinical trials, individuals are enrolled under a reductionist approach that assumes the individuals have a common phenotype (i.e. elevated blood pressure), and are treated following algorithms derived from large population studies [36]. The trial results are described as the median blood pressure in a given treatment arm, which is characterized by a therapeutic regimen that does not necessary distinguish among subtypes of patients. This ‘one size fits all’ approach is expensive and is not adapted to current biomedical research, which explores differences in disease presentation and response to therapies among individuals [55], rather it may oversimplify the complex nature of most diseases. Therefore, with this approach based on the conventional diagnostic paradigm, the descriptions of the disease become broad, focusing less on individual diversities in terms of heterogeneity in the pathophysiology, and the underlying mechanisms of the symptoms. Hence, the characterization and distinction among multiple diseases with shared symptoms are elusive [55–57]. On the other hand, a more personalized approach based on the specific disease pathology, symptoms, signs, and response to treatment of the individual patient would improve diagnosis and risk stratification. It would also identify new pathophysiological pathways, tailor the appropriate therapy in order to improve adherence, and take into account that blood pressure levels and target organ damage may differ among people, not only as a consequence of different exposures to environmental factors but also because of genetic variation in susceptibility to develop disease in response to the environment.

Major clinical guidelines, while advocating evidence-based suggestions, are not highly personalized to the pathophysiology of individual patients and fundamentally use a standard universal approach at the individual level [10]. Thus, taking into account that patients with hypertension may have a different genetic predisposition, as well as the underlying mechanisms for high blood pressure may differ among individual patients, a more personalized and different approach to the treatment of hypertension warrants consideration in order to improve the detection, evaluation, treatment as well as prevention of hypertension, and eventually to improve adherence. Therefore, the large variety of antihypertensive drug options may require personalization for individual patients and a careful balance of antihypertensive efficacy, indications, and contraindications, in order to predict which drug will lower blood pressure most effectively in an individual subject with a good cost-effectiveness profile.

In this review, we discuss the fundamentals of personalized medicine for the management of chronic diseases such as hypertensive cardiovascular disease, focusing particularly on the possible applications of this new paradigm for the evaluation and treatment of hypertensive patients. The ultimate aim is to provide the physician with a precise tool to identify emerging biomarkers that will better address the therapeutic choice to improve the adherence to antihypertensive therapies and reach better blood pressure control in the individual patient and population at large.

## Rationale of the personalized approach to hypertension: former approaches and new opportunities

The pathophysiology of hypertension is characterized by a complex interplay between susceptibility genes, physiological systems, and environmental factors, which develop over time [6]. Several methods of personalized treatment of hypertensive patients have been proposed and investigated. Personalized therapy in hypertension is conditioned by the recognition of a group of traits that may differentiate the response of individual patients. Thus, a modern approach to personalized treatment should consider the use of diagnostic and screening methods that take into account the distinctive molecular or risk profile of the individual patient in order to better identify the predisposition toward a disease or personalized management of the disease.

### Stratifying patients based on renin profiling

The role of plasma renin activity (PRA) in the regulation of blood pressure and response to medications has been explored over many decades [58], and enormous effort was spent stratifying patients according to their renin, sodium, and volume profiling [59]. The approach using renin profile-guided treatment was characterized by equal or better blood pressure control compared with clinical decisions not informed by PRA [60], and represented the starting approach to physiological phenotyping that laid the foundation for hypertension precision medicine. However, the clinical utility of renin profiling has been questioned due to the influence of several confounders, such as sex, race, previous drug treatment, and assay issues, which might reduce the power of this approach in stratifying hypertensive patients. Nevertheless, the ratio between PRA and aldosterone is currently used to make a diagnosis of primary aldosteronism, although is not recommended by guidelines for widespread screening in hypertensive patients. Measuring circulating plasma renin in patients can also indicate the contribution of salt sensitivity to the disease. A low plasma renin can be considered a very specific marker for Na^+^ excess. This can address the therapeutic choice toward diuretics rather than other therapeutic options, particularly in individuals of African origin who have a salt-sensitive type of hypertension that is more responsive to diuretics or calcium channel blockers. In this regard, ethnicity can also be used to stratify treatment in an individualized perspective [61]. For instance, patients with Caucasian background younger than 55 years of age are more responsive to angiotensin-converting enzyme inhibitors (ACEIs) or angiotensin receptor blockers (ARBs) reflecting an overactive renin angiotensin aldosterone system.

### Former single-nucleotide polymorphism studies

Several genetic single-nucleotide polymorphisms (SNP) have been investigated as potential predictors of antihypertensive drug response, particularly in rare monogenic syndromes of hypertension secondary to the disruption of a specific pathway [62]. For instance, in Liddle’s syndrome (an autosomal dominant condition with hypertension associated with suppressed aldosterone and renin levels), a mutation in the epithelial Na^+^ channel gene induces increased rates of Na^+^ reabsorption, volume expansion, and hypertension. In this condition, specific inhibitors of the epithelial Na^+^ channel, such as amiloride or triamterene, could control elevated blood pressure values. Another example is familial hyperaldosteronism type 1, which is an autosomal dominant syndrome characterized by increased aldosterone secretion in response to pituitary adrenocorticotropic hormone (ACTH), which is responsible for hypertension. Individuals with this mutation respond to glucocorticoids, via the suppression of pituitary ACTH secretion [62]. A particular insertion/deletion polymorphism in the *ACE* gene was studied in a large population with hypertension, in order to examine the relationship with treatment response and coronary heart disease. The results demonstrated that there was no effect of insertion/deletion polymorphism on treatment response or coronary heart disease [63].

Although many other SNP-association studies have been performed on hypertension, these studies had some limitations with respect to reproducibility and possible bias [64]; thus, this mode of scientific investigation has largely been rejected. However, the improvement in high-throughput screening, the growing interest in genome-wide association studies (GWAS), as well as advances in proteomic, transcriptomic, and metabolomic technologies, have provided opportunities for stratification by individual genomic and molecular variants in order to define markers that might potentially help target specific defects in the pathways even in essential hypertension, which does not recognize any single known cause.

## Introducing a new paradigm for cardiovascular prevention and therapy—systems biology

Any method used to date for the detection, evaluation, prevention, and treatment of hypertension or its target organ complications is unlikely to be equally successful in all individuals. Strategies tailored to the particular characteristics of individual patients are appealing and might lead to improved health of populations by optimizing outcomes for each individual patient [65,66].

**Figure 1:**
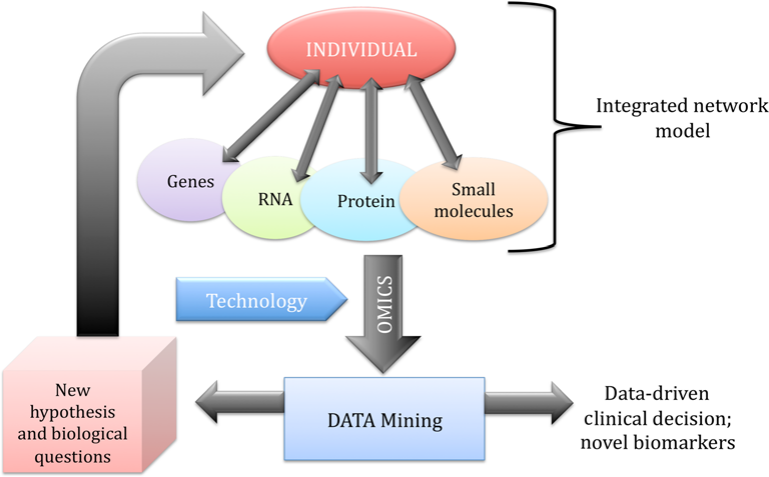
Systems biology integrated network Omics data contribute to find biomarkers of the disease that contribute to clinical decision-making and may induce new hypothesis and biological questions to be tested.

In order to fully understand the rationale and hence the impact of precision medicine, it is important to know the fundamentals of systems biology [57,67]. Systems biology is the study of systems of biological components (i.e. molecules, cells, organisms, or entire species) via the computational and mathematical modeling of complex biological systems, and it is emerging as crucial to all areas of biology and medicine. Systems biology may be considered a ‘holistic’ approach to study the complexity of biological systems. From a biological perspective, organisms (living systems) are considered dynamic and complex, and consist of many integrated networks that communicate at different levels (genome, molecules, cells, and ultimately the entire organism which interacts with the environment) and their behaviors may be complicated to predict exclusively by analyzing the function of individual parts. In other words, a living organism may be considered a ‘network of networks’; thus systems biology focuses on the integrated analysis of the dynamic behaviors of these networks at different levels in order to formulate hypotheses for biological function and dynamical changes (Figure 1). This is different from traditional approaches to the study of living systems, which usually focus on a single scale with a limited understanding of the system. Fundamental to systems biology is the development and availability of modern high-throughput technology such as genomics, proteomics, metabolomics, bioinformatics, and computational models. These provide insights into new pathways and networks between systems, capture real-time molecular phenotypes, drive innovation in biology-based technology and computation in order to discover new biomarkers for disease, which allow the detection of network perturbations foreshadowing the actual development of clinical symptoms, and eventually enable patient stratification on the basis of their individual genetic and molecular profiles (Figure 1). Moreover, this approach may help to understand the determinants of responses to treatments and individualize more specific and personalized targets for therapies by distinguishing in advance those patients that are most likely to benefit from a given drug from those who will suffer side effects (Figure 1). In other words, personalized medicine can be considered as an extension of traditional approaches to the understanding and management of disease, and incorporates individual genetic, molecular, and environmental variability by using *in vitro* diagnostics or imaging technologies (i.e. electroencephalography, electrocardiography, or diagnostic imaging tools) in order to develop accurate and reliable diagnostic tools and predictive biomarkers for the assessment of the patient’s individual characteristics (Figure 1). Thus, the new paradigm is shifting from the study of the ‘average’ cell to the ‘discrete’ cell-subtype in a cell population, as well as from ‘the average’ patient in a global population, to the evaluation of the unique characteristics of the individual patient. Compared with the traditional ‘trial-and-error’ approach to disease treatment, precision medicine, using tools that are more precise, may ensure simplification and speeding up of the diagnostic and decision-making processes and lead to more successful outcomes. Numerous molecular screening strategies have previously been developed for the risk stratification of individuals in order to develop markers capable of detecting the disease at the early phase of its development and to facilitate targeted strategies to prevent pathological complications [61,65,66–68]. For instance, in cancer research, considerable effort is currently spent on identifying the specific molecular markers in a cluster of malignant cells in order to develop specific treatments [69]. Current cardiology practice also relies on a variety of biomarkers, imaging, and clinical information for primary and secondary prevention of cardiovascular diseases, although those markers are not highly personalized. On the other hand, genetic tests and modern transcriptomic, proteomic, and metabolomic methodologies are able to acquire and give information related to the individual and environmental interactions, and may be extremely useful in identifying molecular fingerprints for a personalized approach to guide therapeutic decisions for cardiovascular patients in general and hypertensive patients in particular [66–70].

**Figure 2:**
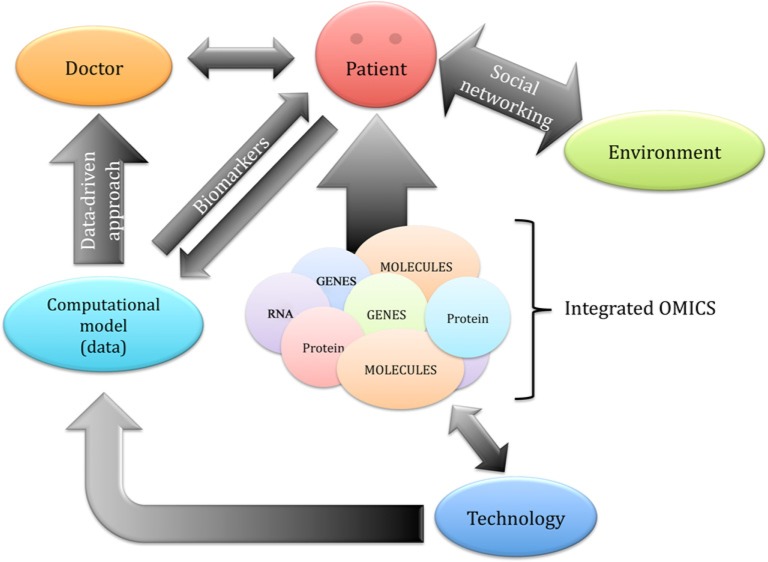
Precision medicine integrated model. Medical doctors and patients are active parts of the integrated processes.

One important aspect of systems biology, and therefore of precision medicine, is data mining, taking into account the massive amount of data generated with current high-throughput technologies [65,66,71] (Figure 1). In order to explore and clinically interpret the complex interactions within the networks that result in individual phenotypes, the availability of advanced computational and analytical techniques, interoperable devices, and the development of informatic systems for the analysis and storage of the big data pool of ‘omics’ (genome sequences and molecular information) in open-to-public databases is crucial [72]. A central role for this task could be played by physicians that can mine data with appropriate algorithms in order to make open-to-public databases that could help data-driven medical decisions (Figure 2). In this process, individual patients also become central stakeholders who contribute to the collection of data by actively participating in shared decision-making (Figure 2). This novel approach could allow better patient monitoring and treatment even outside clinics. Therefore, more people can be treated in their home environment, improving their lifestyle and quality of life. Thus, ‘omics’ technologies have the potential to transform medicine from traditional symptom-oriented diagnosis and treatment toward data-driven disease prevention, early diagnostics, and individualized treatment. There are different examples of the use of currently available high-throughput databases of genome-wide association data obtained from individuals at cardiovascular risk [73,74,75]. For instance, candidate genes for type 2 diabetes (including *CD44* as the top candidate gene) have been identified by combining the results of 130 functional microarray experiments for this disease [74]. Moreover, it has been reported that risk alleles for diabetes were unequally distributed across different human populations, with the risk higher in African populations, by analyzing data of 2510 individuals from 74 populations [75].

Hence, decisions in precision medicine are based on information that integrates data from clinical research, systems biology, and laboratory tests (i.e. molecular and ‘omics’ data), imaging findings, and electronic health records (Figure 2). In this regard, healthcare will become a more dynamic and integrated system, in which the fulfillment of personalized medicine will involve a broad community working together in order to improve patient care by connecting new ideas and inputs in science and technology. As a result, this could improve compliance and clinical outcome, particularly in cardiovascular patients.

## Integrative ‘omics’ as predictive, diagnostic, and prognostic biomarkers in disease-oriented cardiovascular medicine

Currently, molecular disease analyses using a large-scale approach are being employed by some clinicians and pathologists [61,65,66,70] in order to highlight personalized disease susceptibility assessment and thus, patients can be treated according to their own genetic and molecular phenotype.

Whole genome sequencing (WGS) and whole exome sequencing (WES), the single-base analysis of a genome–exome, have become largely available and affordable for genomic studies [61,65,66,70]. Moreover, the availability of next-generation sequencing [76], as well as proteomics [77], metabolomics [78], transcriptomics, and epigenetics [79] stimulated interest in the application of other ‘omics’ to study chronic diseases [80] from an individual and integrated perspective. The network of genetic variants that account for the biological differences between individuals may physically and functionally connect the molecular elements in the system at different levels (i.e. metabolic and/or environmental level) to produce a highly individualized disease phenotype. This novel clinical integrated approach can help to identify perturbations in the system derived from these interactions and to discover specific therapeutic targets that can modulate the key junctions in the integrated networks to more precisely and safely restore the homeostasis of the system and the phenotype [81–83].

### Genomics and pharmacogenomics

Analyzing the genome can be used to discover pathophysiological pathways, evaluate the individual risk for a given person compared with the population at risk (also taking into account the same group of ethnicity, age, and gender), and may suggest potential targets for more individualized therapies. Whole genome sequence revealed variants for both high-penetrance Mendelian disorders [84,85], as well as, complex, chronic diseases including hypertension [86]. Chronic disease-related research has gained significant benefit from WGS and WES technologies [66]. Advanced application of this approach is the use of next-generation WGS and computational genomics in the context of family pedigrees, examining the diversity of the human genome over the entirety of the human population. Family genomics is a powerful and precise method for identifying sequencing errors, disease-causing gene mutations, and genetic relationships between individuals, as has been applied to identify high-risk genes for diseases such as familial thrombophilia, obesity, and psoriasis [87].

Currently, GWAS studies have reported the association of more than 7000 SNP with over 700 complex traits [88]. The study of complex traits may include a variety of chronic diseases ranging from cancers to other chronic complex diseases including diabetes, hypertension, and cardiovascular diseases. A large number of cancer genomes have been sequenced, for different types of tumors [89–100], which may help to understand the susceptibility to the disease as well as better addressing treatment. In addition, for cardiovascular disease, several studies have been performed for genetic associations in individual patients in order to test the risk of developing the disease. For instance, genomic analysis for the risk of cardiovascular diseases was estimated in healthy subjects demonstrating increased probability of risk for myocardial infarction and coronary artery disease [101]. In patients with a family history of vascular disease and early sudden death, genetic variants associated with heart-related morbidities as well as drug response were identified [102]. This information could facilitate future healthcare for an individual patient. In hypertension research, GWAS studies have discovered approximately 63 loci influencing blood pressure, although these variants are responsible for less than 1% of variation in blood pressure in the general population [61,88]. In individuals with hypertension in the Framingham Heart Study population, genetic variants that led to severe hypertensive phenotypes or even protection against hypertension were identified [103]. Some of these common variants are correlated to pathways that can be targeted for individualized therapy. Indeed, it is known that the same drugs may have different effects on different individuals due to their personal genomic background and living habits [104,105], as genetic polymorphisms may partially account for interindividual variability and lack of consistent responsiveness to antihypertensive drugs.

The identification of key enzymes that may play a role in variation in drug metabolism and response has provided the basis for pharmacogenomics. This critically important area of personalized medicine specifically focuses on the variations of DNA and RNA characteristics related to individual drug response [104,105], and may suggest tailored antihypertensive therapeutic options in order to reduce side effects and ultimately healthcare costs [88].

Several hypertension studies have focused on genetic association with the effect of different antihypertensive drugs, particularly diuretics and β-blockers. For instance, a previous GWAS study identified a single SNP in the upstream end of the uromodulin gene (*UMOD*) associated with blood pressure regulation as well as hypertension [106]. The *UMOD* gene is expressed in the kidney at the thick ascending limb of the loop of Henle and may interact with the cotransporter 2 (NKCC2) channel modulating the reabsorption of filtered Na^+^. Thus, in patients carrying this mutation, furosemide could be the first-line therapeutic choice since it is an inhibitor of NKCC2. Yet genotype-directed trials are required to determine whether the *UMOD* variant could individualize patients that potentially gain benefit from loop diuretic treatment, particularly in the setting of uncontrolled hypertension. Furthermore, the genetic polymorphism rs4149601G>A of the *NEDD4L* (which encodes the NEDD4 protein that controls the cell surface expression of different sodium transporters including ENaC and NKCC2) has been associated with reduced ENaC expression, sodium retention, and hypertension with lower plasma renin activity [107–110]. Thus, patients carrying this mutation could better respond to a thiazide diuretic as shown in different clinical studies [111–113]. In the Pharmacogenomic Evaluation of Antihypertensive Responses (PEAR) study only hydrochlorothiazide showed a greater blood pressure lowering effect in patients with GG alleles [112]. In the Nordic Diltiazem (NORDIL) study, carriers of the G allele exhibited a better blood pressure lowering effect and better outcome in response to the thiazide diuretic/β-blocker combination treatment than patients with AA genotype [111]. Consistently, in the International Verapamil SR Trandolapril Study (INVEST) patients who carried the G allele showed cardiovascular risk if not treated with a thiazide diuretic [112]. Another GWAS study in over 60,000 subjects identified a polymorphism of *ADRB1* associated with hypertension and the response to β-blockers. This gene encodes the β-1 adrenergic receptor for the catecholamines, and the Arg389 and Ser49/Arg389 haplotypes have been associated with greater response to β-blockers [114–117]. Interestingly, in the INVEST study, patients with these aplotypes presented increased cardiovascular risk on verapamil therapy alone, the risk was offset by using β-blockers. Nonetheless, future researches need to better define whether these polymorphisms could be used to guide therapeutic decision in hypertensive subjects.

The advances in epigenomics have provided another strategy for defining subgroups of hypertensive patients who might benefit from specific therapies. For instance, aldosterone may target the epigenetically modified sodium channel epithelial 1α subunit (*SCNN1A*), which has been demonstrated to present hypermethylation of histone H3 at lysine 79 (H3K79) at subregions of the promoter in some individuals [118]. The effort of identifying functional genetic and epigenetic markers is extremely important to identify a new category of mechanism-based genetic biomarkers that can facilitate the management of hypertension [119] from an individualized perspective.

In spite of these advances, there is still no ideal biomarker available with a high predictive accuracy for the response to antihypertensive therapies. Therefore, prospective randomized studies are required in order to detect biomarkers and demonstrate a clear clinical utility of the genomic approach [120–122], in terms of improved outcomes. Recently, this approach was employed in a large Chinese population in which 11 biomarkers were used to predict the development of hypertension with some success and accuracy [123]. The GENRES randomized, placebo-controlled, cross-over trial [121] has also suggested a possible relationship between nephrosis (*NPNS1*) gene variants and response to the angiotensin receptor blocker losartan, as well as between PRKCA (which encodes protein kinase Cα) gene variants and blood pressure response to hydrochlorothiazide.

Moreover, experimental studies have demonstrated that a p38 mitogen-activated protein kinase (MAPK) inhibitor suppressed the markers of end-organ damage, osteopontin, and plasminogen activator inhibitor 1, in a rat model of hypertension and correlated with improved end-organ function [124].

On the other hand, genetic studies present some limitations [125] related to the fact that they typically only analyze a small fraction of the genome, in addition, gene–gene interactions are largely neglected. Moreover, genomic information alone is not usually adequate to predict disease onset, and other factors such as environment are expected to play a critical role in the pathophysiological process [126,127], particularly for alterations in blood pressure regulation.

### Other personalized ‘omics’ in precision medicine

Other ‘omics’ technologies might also have an impact on personalized medicine providing increasingly detailed and individualized characterization of hypertension subgroups. Among them, proteomics, transcriptomics, and metabolomics may contribute to defining the fingerprints and markers that constitute the foundation for precision medicine. The profiles of the transcriptome, proteome, and metabolome are more precise indicators of the real-time phenotype, compared with genomic sequences alone; therefore, collecting this ‘omics’ information in an integrated manner would allow monitoring the physiological state of the individual patient in a more complete way, which could complement the systems biology approach, particularly for a multifactorial and complex disease such as hypertension.

Proteomics is defined as the characterization and quantification of proteins in an organism. The information to build every protein in an organism is contained in the DNA, but not every protein is produced at once or in the same amount. The current use of mass spectrometry has enabled great advancements in the proteomic analysis of biological macromolecules with high sensitivity and accuracy [128,129]. With this technology it is possible to quantify a large amount of proteins in a single sample, while simultaneously detecting the expression of mutations and editing events in the human proteome [119], as well as profiling of the phosphoproteome [130]. High-throughput sequencing technologies have also enabled whole transcriptome (cDNA) sequencing [131], which is a powerful tool for disease-related studies, as it can reflect the actual gene activity by also detecting splicing isoforms [132]. Combining such information with genomic information may be valuable in the treatment of chronic diseases, including hypertension.

Profiling small molecules (i.e. metabolome) in a comprehensive and quantitative manner in biological fluids also provides an analysis of multiple biological pathways [70,133]. Since the metabolome reflects the metabolism as well as the real-time energy status of the living organism, it is expected that certain metabolome profiles may provide mechanistic insight and might be associated with different diseases [134]. Particularly fascinating is that it could result in the metabolomic analysis of hypertension, since hypertension is often associated with alteration of metabolic pathways. Therefore, metabolomic profiles are an important aspect for personalized medicine [135,136], predominantly for cardiovascular disease. In particular, it has been shown that the atenolol-induced changes in the metabolome are dependent on race and genotype, which can help to explain the differential metabolomics signature of response to atenolol and the differential response in blood pressure to β-blockers [137].

## Challenges, opportunities, and future directions

Precision medicine is an emerging and growing field, and will probably be of major importance in the future. Growth in this area has been facilitated by the development of systems biology and high-throughput technologies. Undoubtedly, the increasing knowledge and interpretation of personalized ‘omics’ data will enhance our understanding of physiological events during health and disease, and promote personalized diagnosis and treatment. Using this approach, precision medicine aims to reduce the burden of disease by targeting prevention and treatment more effectively through the integration of inputs from multiple data sources. Moreover, individualized medicine might also decrease healthcare costs and prevent adverse events by improving the ability to select the right therapy at the right time for an individual patient.

However, personalization will become more difficult in hypertension since it needs to integrate biological, nonbiological, and environmental factors in decision-making, much more than other conditions such as cancer therapy where genomic factors may play a central role. In complex diseases, such as hypertension, peripheral components of the biological network would be more likely to contribute to the disease than genetic determinants, which are also more probabilistic than deterministic for the development of the disease. Thus, in hypertension, genetic or molecular markers cannot be considered isolated from other reasons for uncontrolled hypertension, which include salt sensitivity, excessive alcohol consumption, sleep apnea, renal artery stenosis, and comorbidities such as obesity, diabetes, and chronic kidney disease. Furthermore, the intraindividual variability in blood pressure levels may also have an impact on the determination of response to drugs. Finally, the impact of environment and personal choices also affects patient behavior and treatment outcomes.

Thus, future approaches to the personalization of hypertension treatment will possibly focus less on the DNA sequence and more on variables that can change over time. The fields of epigenomics, metabolomics, proteomics, and transcriptomics are likely to yield data useful in personalizing the treatment of hypertension. This combined information not only determines the genetic susceptibility of the person, but also monitors his/her real-time physiological states [67].

Personalized medicine, using ‘omics’ approaches, relies on the growing development of technology for biological research, which is now affordable due to the rapid drop in technology costs. A challenge that should not be overlooked in this new era of complex data regards storage and the analytical aspect of the ‘omics’ data, and as such it will be critical for biologists, clinicians, bioinformaticians, and data scientists to work together in a truly integrated manner to ensure the continued improvement of this field.

**Figure 3:**
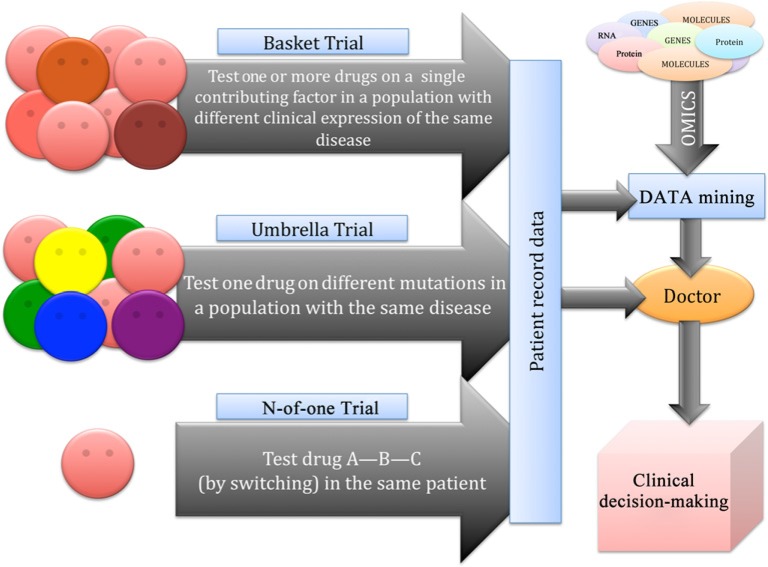
Different types of trials for personalized medicine. Characteristics of Basket trial, Umbrella trial, and N-of-1 trial.

However, the significance of any specific approach to personalized medicine still needs to be tested. Therefore, any proposed personalization method would theoretically need a clinical trial to demonstrate benefit above current guideline-based therapy, although less rigorous evidence might be acceptable [138]. In addition to traditional types of clinical trials, such as randomized, cross-over, double-blind etc., several new approaches for phase II clinical trials have been developed, such as *Basket* and *Umbrella* trials, as well as the N-of-1 trial that might better account for variability between patients [139–142] (Figure 3). In particular, in *Basket Trials*, drugs are tested based on their mode of action and all participants included in the trial have a common contributing or facilitating factor, such as an abnormal protein or metabolic pathway, which may be the target of the drug. In *Umbrella Trials*, the opposite strategy is followed. Participants present the same clinical diagnosis but specific genetic markers or other factors may vary. Drugs are tested according to individual profiles, therefore in one trial several different drugs may be tested for the same disease, and adaptive trial protocols that allow modifications to interventions based on participant responses for a subset of individual participants are allowed while the trial is ongoing. However, these studies are not considered sufficiently personalized. Therefore, a trial centered more on individual (such as N-of-1 trials) than on ‘average responses to therapy’ is required. N-of-1 trials have already been utilized as a matter of necessity for rare diseases. In this type of trial, different treatments (including placebo) are evaluated in one patient over a period of time. Although the N-of-1 trial may require elaborate study design and the results may not be easily extrapolated to other patients, it can provide useful medical information for subsets of patients in the general population. A detailed discussion on clinical trials informed by biomarkers has recently been reviewed by Antman and Loscalzo [65], and a critical discussion on the new types of clinical trials has been reviewed by Schork [139]. It is now timely to extend clinical trials beyond the classical clinical approaches, by considering methods to address personalized medicine. This requires specialized centers for the application of high-throughput system biology technology as well as resources and infrastructure to collect integrated computational data. This implies that future healthcare systems will need the integration, analysis, and representation of data to enable data visualization and a more precise selection of therapeutic regimens [143].

A discussion that warrants some consideration relates to the economics and cost-benefits associated with personalized medicine. As the field evolves and as ‘large data’ results become available, comprehensive health economic analysis will be crucial. To date, we are still in the infancy phase of personalized medicine and hence economic analysis is based on predictions and theoretical knowledge. If personalized medicine does lead to the ‘right medicine for the right patient at the right time’ then health management and associated costs should be less than the current state, where the economic burden of treating cardiovascular disease is enormous. It should also be highlighted that the costs of ‘high-throughput technologies’ to profile individual patients have been markedly reduced over the past decade and hence global expenses to invest in this new approach should be more affordable, would be worthy, and might contribute to reducing general healthcare costs, since the global cardiovascular risk could be controlled better in numerous patients in primary and/or secondary prevention, and, optimistically, personalized medicine would contribute greatly to reducing the burden of cardiovascular events which is currently still high and contributes substantially to healthcare expenses.

Furthermore, the new approach toward personalized medicine involves the patient as a central and active participant of the complex network. Education and training for healthcare providers and patients are essential to prepare for the next phase of modern medicine so that the tremendous potential of personalized medicine, for the individual patient and ultimately the population at large, can be realized.

## Abbreviations

ACEI: angiotensin-converting enzyme inhibitor
ACTH: adrenocorticotropic hormone
ARB: angiotensin receptor blocker
GWAS: genome-wide association
MAPK: mitogen-activated protein kinase
SNP: single-nucleotide polymorphisms
WES: whole exome sequencing
WGS: whole genome sequencing
